# Prevalence of occult hepatitis C virus infection in hemodialysis patients

**DOI:** 10.22088/cjim.12.4.533

**Published:** 2021

**Authors:** Masomeh Bayani, Mohammad Reza Hasanjani Roushan, Mohammad Jafar Soleimani Amiri, Yousef Yahyapour, Soheil Ebrahimpour, Somayeh Akbarzadeh Jelodar

**Affiliations:** 1 Infectious Diseases and Tropical Medicine Research Center, Health Research Institute, Babol University of Medical Sciences, Babol, Iran

**Keywords:** Hemodialysis, Occult infection, Hepatitis C

## Abstract

**Background:**

Via hemodialysis, viral infections can be transmitted in patients a new definition of this infection with no increase in liver enzymes, negative HCV-PCR in serum and presence of virus in the liver and peripheral blood mononuclear cell (PBMC) called occult hepatitis C virus (HCV) infection (OCI). We decided to examine the prevalence of occult hepatitis C infection on hemodialysis cases.

**Methods:**

The current research is a cross-sectional study on patients with end-stage renal disease (ESRD) who were at three hemodialysis centers in Mazandaran province in Iran during 2012-2014. In this study of 356 patients who were undergoing hemodialysis, 54 patients were excluded due to positive HCV Ab, and the remaining 302 patients were enrolled. The test of all serum samples for HCV-RNA detection of plasma and PBMCs was done by real-time polymerase chain reaction (real-time PCR).

**Results:**

There was a significant association between the duration of dialysis with the prevalence of occult HCV infection (P=0.017). Eight (2.65%) patients were positive for HBs Ag and with OCI, but none of them was infected with both hepatitis C and B obviously. Also among the total number of patients, nine patients tested positive for HCV RT-PCR in PBMC in which one of them was positive for serum HCV RNA PCR and was excluded from the study.

**Conclusion:**

The results showed that eight patients had an OCI. There was not any association found between age and sex with OCI, but there was a significant relationship between the duration of dialysis with the prevalence of OCI.

Broader access to dialysis extends the life of many patients with end-stage renal disease (ESRD). Although hemodialysis therapy in patients with ESRD as a way to increase life expectancy in these patients, but there is the increase risk of developing related complications. ESRD is associated with immune system dysfunction (1). In ESRD patients, activated T lymphocyte count is decreased. Thus the function of memory T cells is diminished in ESRD (2). ESRD patients are very susceptible to viral infections and reactivate latent infections. Some infections can be transmitted through hemodialysis from infected patients to non-infected patients. Among these viral infections that can be transmitted in these patients, hepatitis B, hepatitis C and HIV are noted (3, 4). Hepatitis C is a liver infection caused by the hepatitis C virus (HCV) is a global health concern. Based on the World Health Organization (WHO) report, approximately 130-210 million people are infected with HCV (5). Researchers conducted on the general population of Iran found the estimated HCV infection prevalence rate to be 0.3% to 1.6% (6, 7). Hepatitis C is a viral disease that primarily affects the liver and a large proportion of cases become chronic, consequences developed to liver cirrhosis (27%) and hepatocellular carcinoma (25%) (8-12).

One of the main ways of transmission of this disease is transmitted through blood and blood products. People who are infected with HCV through blood transfusion are patients who require blood transfusion and blood products continually and among them, are the patients with thalassemia and chronic kidney failure who are undergoing hemodialysis. Recently, a new form of HCV infection, has been defined as occult HCV infection (OCI). OCI is characterized by the absence of anti-HCV antibodies and serum HCV RNA but by the presence of HCV RNA in the peripheral blood mononuclear cells (PBMCs) of within 70% of patients and in the liver of all cases (13, 14). The HCV RNA in the PBMCs and normal alanine aminotransferase (ALT) levels years after the resolution of infection has also been detected (15). The established ability of HCV replication in PBMCs raises the question of potential transmission of infection to some cell types, or individuals through blood transfusions and hemodialysis. In fact, the transmission of infection via hemodialysis is feasible. The aim of this study was to conclude the prevalence of OCI in hemodialysis patients.

## Methods

This cross-sectional study was conducted in all patients undergoing hemodialysis in three centers: Shahid Beheshti-Babol, Valiasr-Ghaemshahr and Imam Khomeini –Fereydunkenar in Mazandaran province in northern of Iran between 2012 and 2014. In this study of 356 patients undergoing hemodialysis were (Shahid Beheshti Babol with 167 patients, Vali Asr- Ghaemshahr with 150 patients and Imam Khomeini Fereydunkenar with 39 patients), 54 patients had HCV Ab positive and excluded from the study (Shahid Beheshti Babol center with 24 patients, Vali Asr-Ghaemshahr with 23 patients and Imam Khomeini Fereydunkenar with 7 patients). And finally the remaining 302 patients were enrolled. Whole blood samples (5 ml) were collected in ethylenediaminetetraacetic acid (EDTA) vacutainer tubes from the cases who were negative for HCV and HBV markers and processed about two hours for the separation of plasma and PBMCs. Anti-HCV antibody was detected by a third generation enzyme linked immunosorbent assay (ELISA; HCV 3.0 ELISA Ortho, Raritan, NJ) and HBsAg was determined using commercially available enzyme immunoassays (Abbott GmbH, Wiesbaden-Delkenheim, Germany), according to the manufacturer’s instructions. The study protocol was confirmed by the thics committee of Babol Unversity of Medical Sciences and informed written consent was obtained from individuals.

**Plasma and pbmcs preparation and preservation:** PBMCs were prepared immediately from the EDTA blood by the standard density gradient centrifugation on Ficoll Hypaque. All the reagents used in PBMCs preparation were obtained from Lonza Bio products (Belgium). Plasma was stored in several aliquots at 20°C until further analysis. Aliquots of 5–10º 106viable cells/ml were stored at 80°C in freezing solution (90% fetal bovine serum and 10% Dimethyl sulfoxide, Sigma, Carlsbad, CA) until further analysis.

**RNA extraction from plasma and pbmcs: **The automated extraction of viral RNA was performed from thawed plasma using the QIAamp1 Viral RNA Mini-Kit (cat#1048147, QIAGEN1, Germany) following the manufacturer’s protocol From PBMCs. This was performed by the thawing of PBMCs, followed by automated extraction of total RNA from lysed PBMC pellet using lysis buffer included in the QIAamp1 RNA Blood Mini-Kits (cat#52304, QIAGEN1, Germany) following the producer's guide.

**HCV-RNA detection of plasma and pbmcs by standardized quantitative real-time PCR: **HCV viral load was determined first in the plasma then in PBMCs using Artus1 HCV-RGRT-PCR Kit (cat#4518265, QIAGEN1, Germany)following the manufacturer’s protocol and amplification was carried out on an Applied Biosystems 7500 Fast Real-Time PCR Thermal cycler (Life Technologies, Alexander City, AL). Positive and negative controls were obtained from patients with active chronic HCV infection and healthy volunteers, respectively. Control samples were examined for HCV viral load to determine the sensitivity of our method. Samples were considered positive if they showed at least two positive results in two different reactions.

**Statistical analysis:** Statistical analysis was performed using IBM SPSS statistics 16. Comparison of qualitative variables was performed with x^2^ test. Student’s t-test (or a non-parametric Mann–Whitney U-test when appropriate) was performed for comparisons of continuous data. A p-value <0.05 was investigated statistically significant.

## Results

**Figure 1:**
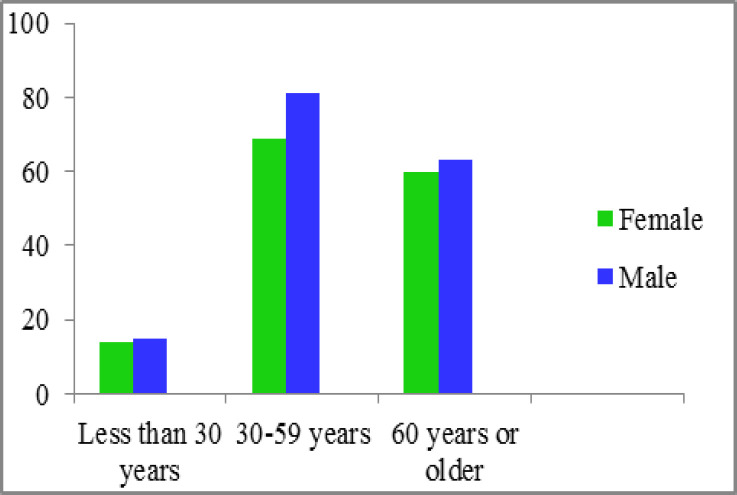
Frequency of different age groups in study participants

**Figure 2:**
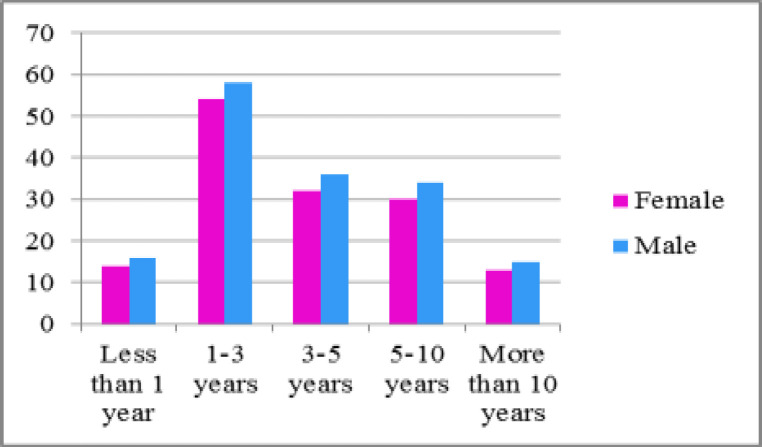
Frequency of Dialysis duration and sex among participants

The findings showed that 8 patients had occult HCV infection. The mean age of patients was 43.46±6.7 with the range of 23-69 years, including 143 (47.4%) women and 159 (52.6%) patients were males (Figure1, 2). The current study revealed that the majority of study participants (29.8%) had a high school education, 5.9% were smokers and 3.6% of them have been taking opium. Seventy six (25.2%) patients had a history of diabetes mellitus and in 72 (23.8%) patients were seen with a history of high blood pressure (Table 1). 

There was a significant association between the duration of dialysis with the prevalence of occult HCV infection (P=0.017) (Table 2). Eight patients were positive for HBs Ag, none of them was infected with both hepatitis C and B. Also among the total patients, 9 patients tested positive for HCV RT-PCR in PBMCs in which one of them was positive for HCV RNA PCR and were excluded from the study and thus the remaining 8 patients (2.65%) had an occult hepatitis C infection.

**Table 1:** Frequency of demographic data on the study participants

**Variables**		**Number**	**Percent(%)**
Age(Years)	<30	29	9.6
	30-59	150	49.7
	>60	123	40.7
Sex	Male	159	52.6
	Female	143	47.4
Education	Illiterate	45	14.9
	Primary	77	25.5
	Guidance	51	16.9
	High school	90	29.8
	Collegiate	39	12.9
Location	City	241	79.8
	Village	61	20.2
Duration of dialysis (years)	<1	30	9.9
	1-3	112	37.1
	3-5	68	22.5
	5-10	64	21.2
	<10	28	9.3
Smoking		18	5.9
Drug use		11	3.6
Alcohol consumption		0	0
DM		76	25.2
HTN		72	23.8

All of the authors would like to thank the staff nurses at the dialysis units of Shahid Beheshti, Vali-e-Asr and Imam Khomeini Hospitals for their help with sample collection.

**Table 2:** Correlation between variables with occult HCV infection

**Variables**		**OCI+** **(N=8)**	**OCI-** **(N=294)**	**P -value**
Mean age		55.7±8.2	59.1±9.4	0.85
Age (years)	<30	0 (0%)	29(9.9%)	0.309
	30-59	6 (75%)	144(49%)	
	>60	2 (25%)	121(41.1%)	
Sex	Male	6 (75%)	137(46.6%)	0.109
	Female	2 (25%)	157(53.4%)	
Education	Illiterate	3(37.5%)	42 (14.3%)	0.088
	Primary	0 (0%)	77 (26.2%)	
	Guidance	3(37.5%)	48 (16.3%)	
	High school	2 (25%)	88 (29.9%)	
	Collegiate	0 (0%)	39 (13.3%)	
Location	City	7(87.5%)	234(79.6%)	0.495
	Village	1(12.5%)	60 (20.4%)	
Duration of dialysis (years)	>1	0 (0%)	28 (9.5%)	0.017
	1-3	0 (0%)	107(36.4%)	
	3-5	1(12.5%)	67 (22.8%)	
	5-10	2 (25%)	64 (21.8%)	
	<10	5(62.5%)	28 (9.5%)	
Smoking	Yes	0 (0%)	18 (6.1%)	0.608
	No	8 (100%)	276(93.9%)	
Drug use	Yes	0 (0%)	11 (3.7%)	0.741
	No	8 (100%)	283(96.3%)	
DM	Yes	1(12.5%)	75 (25.5%)	0.360
	No	7(87.5%)	219(74.5%)	
HTN	Yes	1(12.5%)	71 (24.1%)	0.394

## Discussion

Generally, hemodialysis patients are at higher risk of HBVand HCV infection. Infection with hepatitis B virus and hepatitis C in patients who were treated with hemodialysis is a major problem (16, 17). Hemodialysis patients are at high risk for viral hepatitis infection due to many blood transfusion sessions, prolonged vascular access, and the potential exposure to infected patients. HBV infection is less prevalent than HCV in hemodialysis units (18). In recent years, a new type of HCV infection has been defined as an OCI which spreads around the world and all HCV genotypes are involved in this type of infection. Some groups are the higher risk to occult HCV infection including patients who undergo hemodialysis and patients with cryptogenic liver disease and family members of patients who had OCI (19). Previously occult HCV infection in hemodialysis patients was studied in a small number of studies. In a study by Thongsawat et al., occult HCV infection in a dialysis center in Thailand was studied and found that occult HCV infection among hemodialysis patients in the country was common (21%) (20). Rinonce et al. showed that occult HCV infection was detected in 12.9% of hemodialysis patients in Indonesia (21). They proposeed that severe infection control programs in dialysis units in the country are essential. Yakaryilmaz et al. reported that the prevalence of OCI in hemodialysis cases in Turkish was 4.8 percent (22). 

The prevalence rate of OCI was reported 3.3% in Italy in a population of 276 subjects unselected for the hepatic disease but not on hemodialysis which was very similar to our results (23). The difference between the prevalence of HCV infection in dialysis patients may be due to the prevalence of occult HCV infection in a variety of different countries and different dialysis centers. The overall prevalence of HCV infection in the general population is about 0.5% (1% in men and 0.1% in women) (24). The prevalence of anti-HCV antibodies in hemodialysis patients is higher than the general population (about 11-25%) (25). The recent study has revealed the risk of OCI has increased along with prolongation of dialysis duration. Assarehzadegan in his study found that duration of hemodialysis treatment was significantly associated with positive hepatitis C (26). In a study of Ahmetagić longer duration of dialysis as independent variables were the statistically significant risk factor for hemodialysis patients (27). 

El Amin et al. demonstrated that hepatitis C-positive in serum was associated with the duration of dialysis(28). Mohtasham Amir et al. found that there is significant association between duration of dialysis and increasing risk of HCV infection (29). There is strong evidence that nosocomial transmission of HCV infection poses during hemodialysis. So, the preventing HCV transmission in patients undergoing hemodialysis is the most important plan at the health staff of hemodialysis centers and cleaning dialysis machines. Isolation of patients with HCV to preventing HCV transmission in hemodialysis units is not recommended (30). The prevalence of anti-HCV Ab in the community is high (7.3%). In some studies, patients who are typically diagnosed as negative HCV infection had HCV viral replication in their own PBMC. This data suggests that HCV potentially can be transmitted from apparently healthy individuals (19). Overall, the prevalence of occult HCV infection in some studies (7.3%) compared to the high prevalence of anti-HCV Ab in the general population (15%) is much lower (31).

Thus, further studies should be conducted for healthy subjects with normal liver enzymes and also several phases of infection with HCV. We conclude that the prevalence of occult HCV infection in hemodialysis patients is more than what we haerin report (2.65%) because of such reasons, like as neither examination of occult HCV infection in liver biopsies nor stimulation of PBMCs with mitogens (32).

In conclusion the results showed that eight (2.65%) patients had occult hepatitis C infection. There was not any relationship found between ages, and sex with occult HCV infection. According to the recent results, considering HCV infection even with its low viral load may be associated with an increased risk of progression to cancer. Such studies can be useful in helping to reduce the risk of transmitting infection.
